# Metabolic Profile in Neonatal Pig Hearts

**DOI:** 10.3389/fcvm.2021.763984

**Published:** 2021-10-14

**Authors:** Pengsheng Li, Fan Li, Ling Tang, Wenjing Zhang, Yan Jin, Haiwei Gu, Wuqiang Zhu

**Affiliations:** ^1^Department of Cardiovascular Diseases, Physiology and Biomedical Engineering, Center for Regenerative Medicine, Mayo Clinic Arizona, Scottsdale, AZ, United States; ^2^Laboratory of Regenerative Medicine in Sports Science, School of Physical Education and Sports Science, South China Normal University, Guangzhou, China; ^3^College of Health Solutions, Arizona State University, Phoenix, AZ, United States; ^4^Department of Environmental Health Sciences, Robert Stempel College of Public Health and Social Work, Florida International University, Miami, FL, United States; ^5^Department of Cellular Biology and Pharmacology, Herbert Wertheim College of Medicine, Center for Translational Science, Florida International University, Port St. Lucie, FL, United States

**Keywords:** neonatal, pig, heart, metabolism, metabolomics

## Abstract

We evaluated the metabolic profile in pig hearts at postnatal day 1, 3, 7, and 28 (P1, P3, P7, and P28, respectively) using a targeted liquid chromatography tandem mass spectrometry assay. Our data showed that there is a clear separation of the detected metabolites in P1 vs. P28 hearts. Active anabolisms of nucleotide and proteins were observed in P1 hearts when cardiomyocytes retain high cell cycle activity. However, the active posttranslational protein modification, metabolic switch from glucose to fatty acids, and the reduced ratio of collagen to total protein were observed in P28 hearts when cardiomyocytes withdraw from cell cycle.

## Introduction

Cardiomyocytes in adult mammals possess very limited regenerative potential as a result of cell cycle exit. Myocardium loss after injuries is typically replaced by fibrotic scar. Several lines of evidence have shown that cardiomyocytes in neonatal mice (1) and pigs (2) retain regenerative capacity. We have recently shown that hearts of postnatal day 1 and day 2 pigs can regenerate lost myocardium in response to injury (2). This regeneration is mediated by proliferation of preexisting cardiomyocytes, which does not occur when cardiomyocytes permanently exit cell cycle. However, mechanisms underlying injury-mounted regenerative response especially in large mammals have not been fully understood.

Cardiomyocytes in postnatal mice undergo hypertrophic growth to adapt to increased blood pressure and volume. The process of cardiomyocyte maturation is associated with a cessation from cell cycle and proliferation activities, along with changes of energy metabolism switching from glycolysis to fatty acid oxidative metabolism to allow more efficient ATP production. Prior research has illustrated metabolic state in developing mouse hearts, and showed that metabolism switch is associated with cell cycle cessation (3, 4). However, metabolic changes and signaling pathways mediating cardiomyocyte maturation and cell cycle withdrawal in postnatal pig hearts have not been reported. Therefore, we performed a targeted metabolomic analysis to evaluate the metabolic profile in pig hearts from postnatal day 1 to day 28.

## Materials and Methods

### Animal Protocol

All animal protocols were approved by the Institutional Animal Care and Use Committee of the Mayo Clinic and were performed in accordance with the National Institutes of Health Guide for the Care and Use of Laboratory Animals. Postnatal day 1 (P1), day 3, (P3), day 7 (P7), and day 28 (P28) pigs (Yorkshire-Landrace Cross background) were obtained from certified vendor S&S Farms.

### Tissue Preparation

A small block of heart tissue (~20 mg) was dissected from apex of each heart (*n* = 6 per age, both male and female were included), and was homogenized in 200 μL MeOH:PBS (4:1, v:v). After adding 800 μL MeOH:PBS, lysates were vortexed for 10 s, and stored at −20°C for 30 min. Samples were sonicated in an ice bath for 30 min, followed by a spin-down at 14,000 rpm for 10 min (4°C). A total of 800 μL supernatant was transferred to a new Eppendorf tube and vacuum dried. The pellets were reconstituted with 150 μL solution containing 40% PBS and 60% acetonitrile prior to mass spectrometry (MS) analysis. A quality control (QC) sample was generated by pooling all the study samples.

### Targeted Liquid Chromatography Tandem Mass Spectrometry (LC-MS/MS)

Targeted LC-MS/MS assay was performed as we described before (5). We implemented a pathway-specific LC-MS/MS method that can cover more than 300 metabolites from >35 metabolic pathways. Each sample was injected twice, one injection of 10 μL was for analysis with negative ionization mode and the other injection of 4 μL was used for analysis with positive ionization mode. Both chromatographic separations were performed in hydrophilic interaction chromatography mode on a Waters XBridge BEH Amide column (150 × 2.1 mm, 2.5 μm particle size). The flow rate was set as 0.3 mL/min, auto-sampler temperature was set at 4°C, and the column compartment was kept at 40°C. The mobile phase was composed of Solvents A (10 mM ammonium acetate, 10 mM ammonium hydroxide in a mixed solution containing 95% H_2_O and 5% acetonitrile) and Solvents B (10 mM ammonium acetate, 10 mM ammonium hydroxide in a mixed solution containing 5% H_2_O and 95% acetonitrile). After the initial isocratic elution for 1 min, the percentage of Solvent B was reduced to 40% (*t* = 11 min), and it was maintained at 40% for 4 min (*t* = 15 min). The mass spectrometer is equipped with an electrospray ionization source. Targeted data acquisition was performed in multiple-reaction-monitoring (MRM) mode. The extracted MRM peaks were integrated using Agilent MassHunter Quantitative Data Analysis.

### Western Blot

A small piece (~100 mg) of left ventricular tissue from each pig heart was isolated. Whole cell lysate was generated using the protocol we reported before (6). Protein concentration was measured with a BCA kit (Thermo Fisher Scientific, cat# 89900). Samples were solubilized in sodium dodecyl sulfate (SDS)-polyacrylamide gel electrophoresis (PAGE) loading buffer for 5 min at 95°C and resolved on 10% SDS–PAGE gels. After the electro-transferred from the gel to nitrocellulose (Amersham) membrane, the equal loading was confirmed by Ponceau S staining (Fisher Scientific, Cat# AAJ6074430). Immunoblotting was performed using antibodies recognizing LDHA (Thermo Fisher Scientific, Cat# PA5-27406), LDHB (Thermo Fisher Scientific, Cat# 14824-1-AP). Signal was visualized by Odyssey CLx Infrared Imaging System (LI-COR Biosciences). Western signal was digitized and quantitated using Image Studio Lite Quantification Software.

### Statistical Analysis

**Figure 1:**
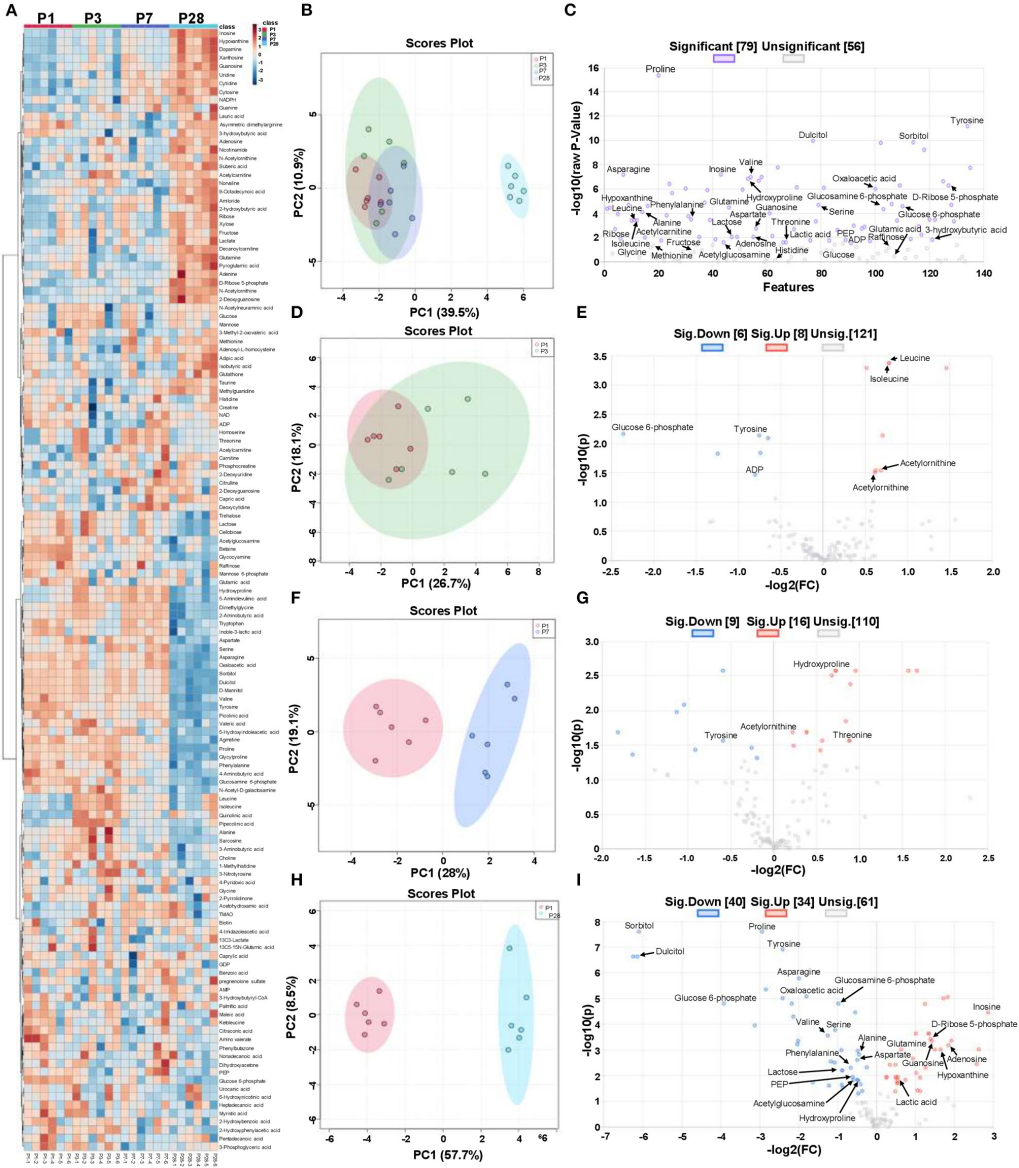
Metabolic state in neonatal pig hearts. Four sets of left ventricular tissue samples were collected on P1, P3, P7, and P28 pigs for LC-MS/MS analysis. **(A)** Heat map of linear mixed effect model estimates for metabolites. **(B)** Principal component analysis (PCA) of metabolites from cardiomyocytes in P1, P3, P7, and P28 hearts. **(C)** Volcano plot of metabolites from P1, P3, P7, and P28 hearts (*p* < 0.05; one-way ANOVA with Fisher's Least Significance Difference Test). The purple and gray dots represented the significant changed and unsignificant changed metabolites. **(D)** PCA of metabolites from cardiomyocytes in P1 and P3 hearts. **(E)** Volcano plot of metabolites from P1 and P3 hearts (*p* < 0.05; Student's *t*-test). **(F)** PCA of metabolites from cardiomyocytes in P1 and P7 hearts. **(G)** Volcano plot of metabolites from P1 and P7 hearts (*p* < 0.05; Student's *t*-test). **(H)** PCA of metabolites from cardiomyocytes in P1 and P28 hearts. **(I)** Volcano plot of metabolites from P1 and P28 hearts (*p* < 0.05, Student's *t*-test). The red, blue, and gray dots represented the up-regulated, down-regulated, and unchanged metabolites, respectively.

**Figure 2:**
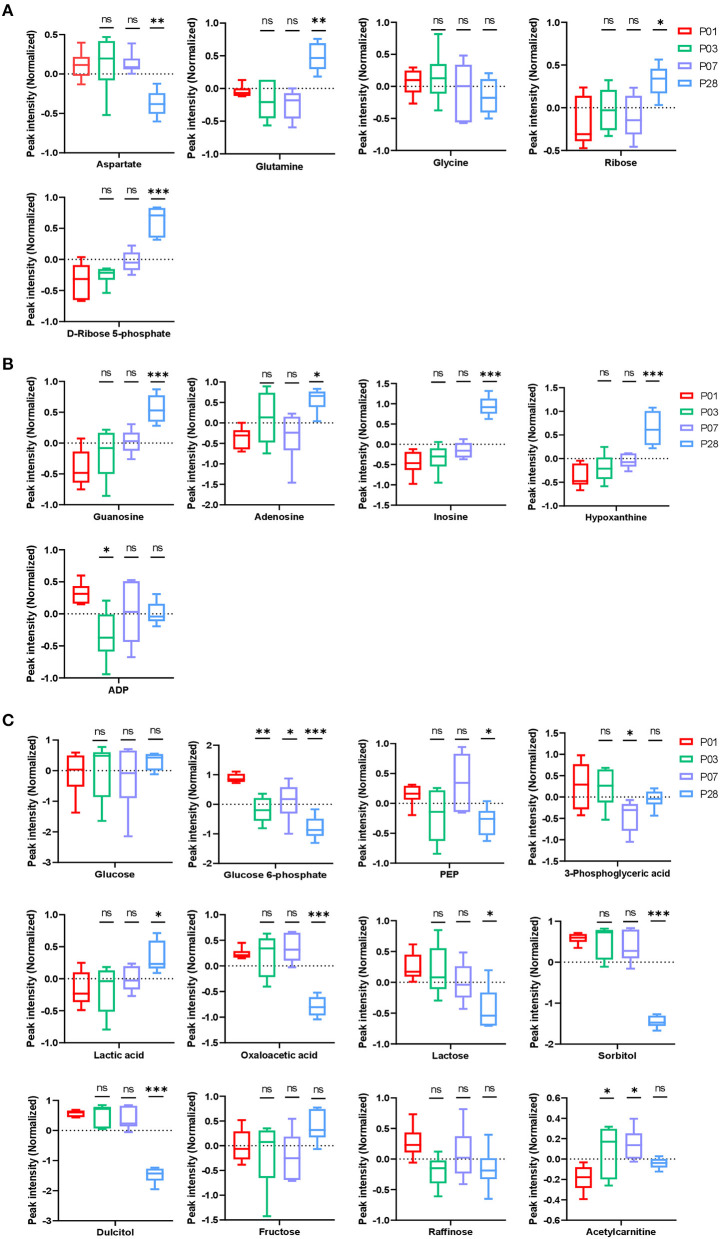
Nucleotide and carbohydrate metabolism in neonatal pig hearts. **(A,B)** Metabolites in the pathway of nucleotide synthesis **(A)** and break down **(B)** in the four sets of heart samples (P1, P3, P7, and P28 hearts). **(C)** Metabolic profile of glucose, other sugars, and fatty acids. *p < 0.05, **p < 0.01, ***p < 0.001, one-way ANOVA with Tukey's Honestly Significant Difference Test. *n* = 6 hearts per age.

**Figure 3:**
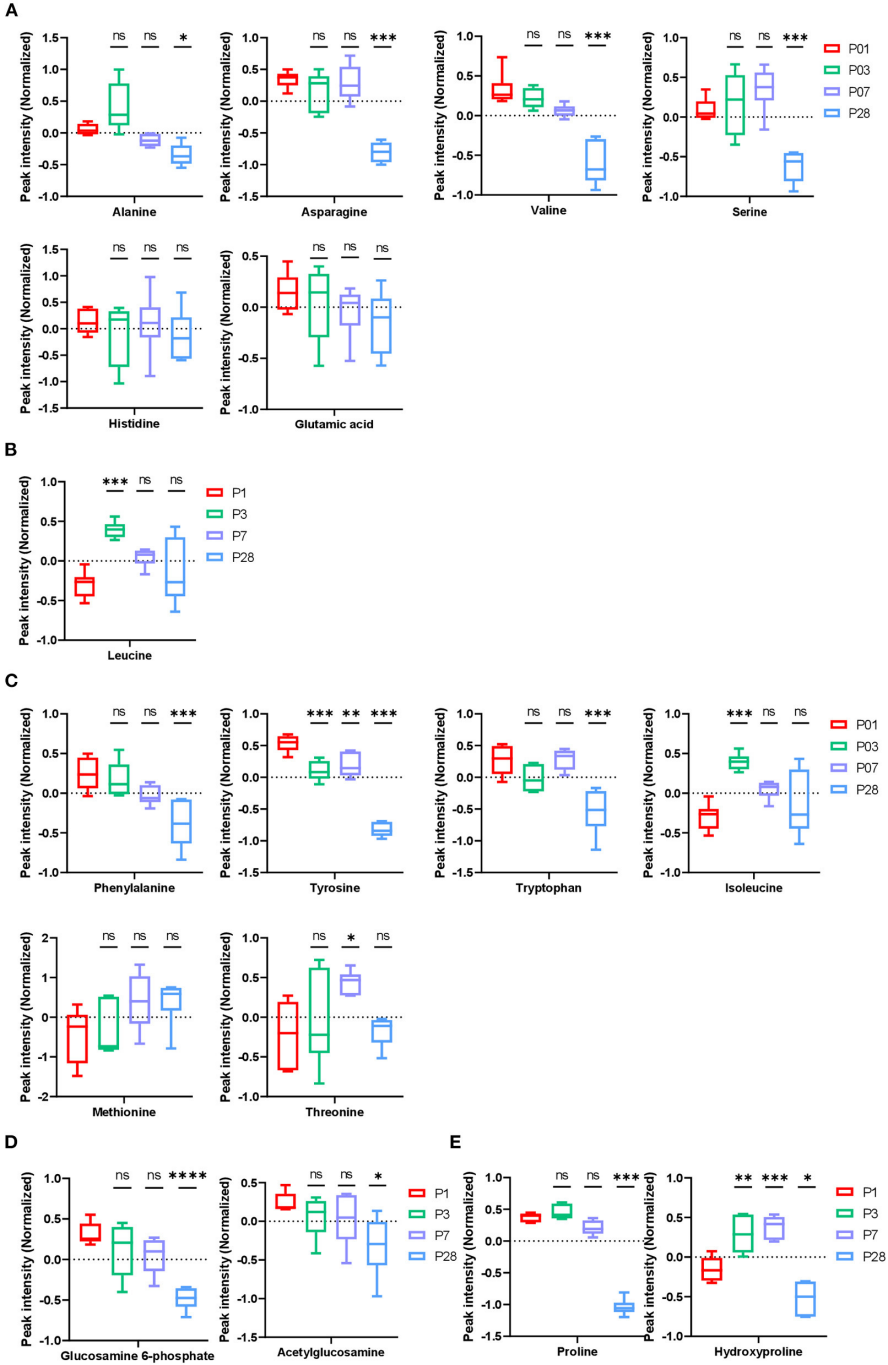
Amino acid and collagen metabolism in neonatal pig hearts. **(A–D)** Metabolic profile of glucogenic amino acids **(A)**, ketogenic amino acids **(B)**, glucogenic/ ketogenic amino acids **(C)**, and glucosamine 6-phosphate **(D)**. **(E)** Metabolites in the collagen synthesis pathway. *p < 0.05, **p < 0.01, ***p < 0.001, ****p < 0.0001, one-way ANOVA with Tukey's Honestly Significant Difference Test. *n* = 6 hearts per age.

**Figure 4:**
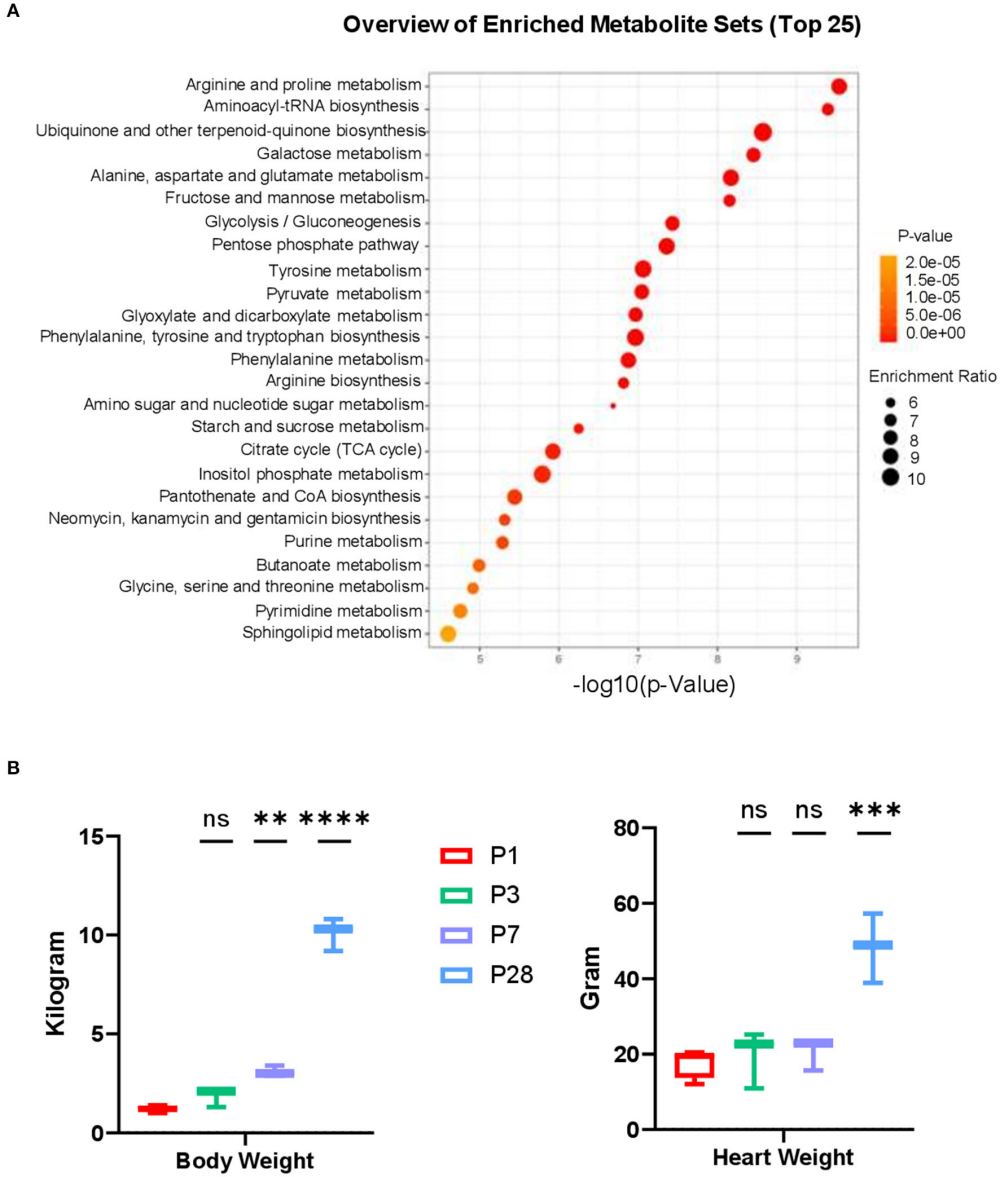
Metabolic pathway analysis and cardiac attributes in neonatal pig hearts. **(A)** Metabolite set enrichment analysis on metabolites in the P1 and P28 hearts. **(B)** Body weight and Heart weight in the P1–P28 animals. **p < 0.01, ***p < 0.001, ****p < 0.0001, one-way ANOVA with Tukey's Honestly Significant Difference Test. *n* = 6 hearts per age.

**Figure 5:**
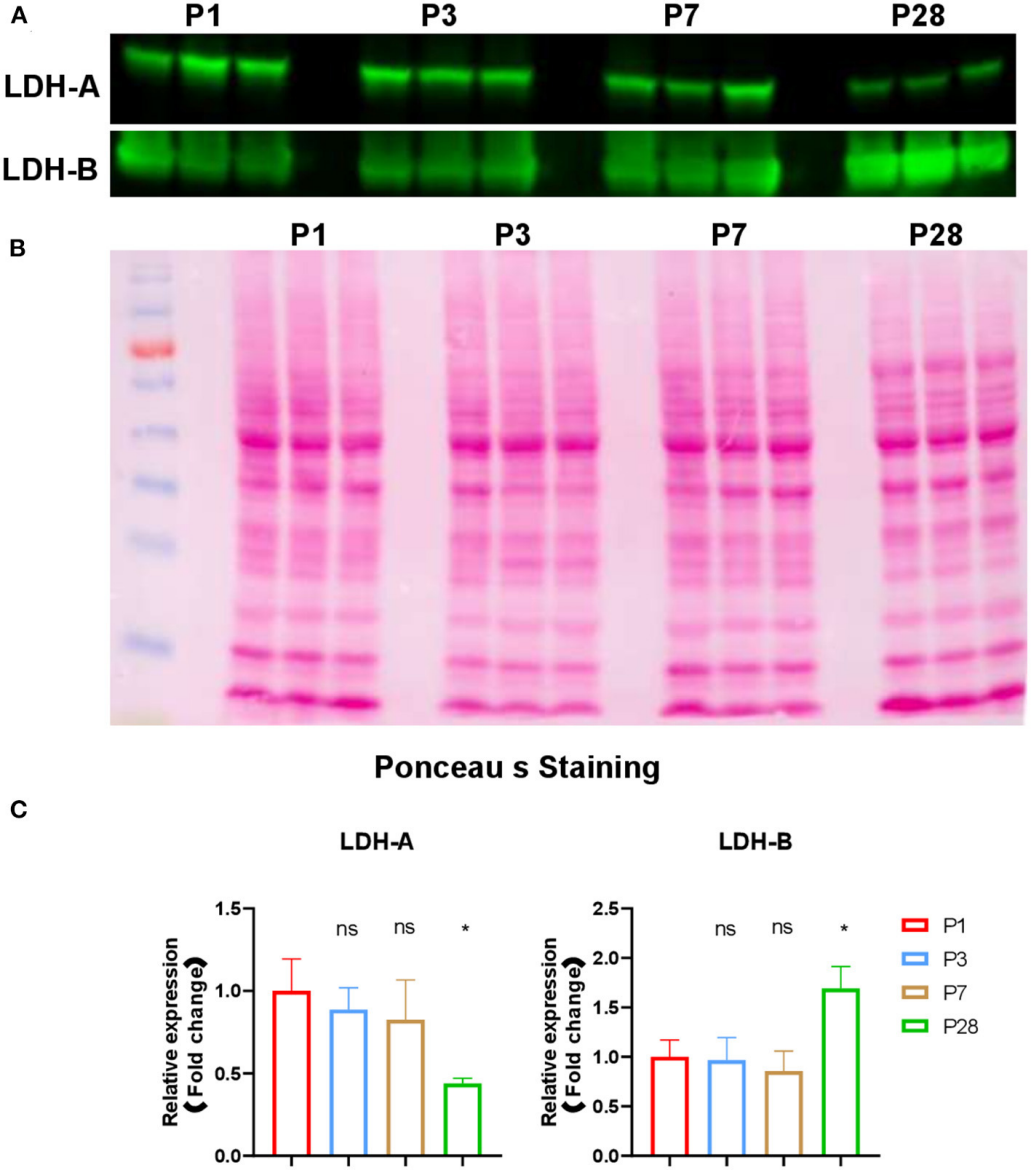
Expression of LDHA and LDHB in neonatal pig hearts. **(A)** Western blot analysis of expression of LDHA and LDHB in in the four sets of heart samples (P1, P3, P7, and P28 hearts, *n* = 3 hearts per age). **(B)** Ponceau S staining of the blot to confirm equal protein loading. **(C)** Quantification of band intensity by western blot via densitometric analysis. *p < 0.05; one-way ANOVA with Tukey's Honestly Significant Difference Test.

All data were log10 transformed, mean-centered, and divided by the square root of standard deviation (SD) for each metabolite (Pareto-scaled) to approximate normal distribution. Data in Figure 1C were processed for univariate statistical testing using one-way ANOVA with Fisher's Least Significance Difference Test. Data in Figures 1E,G,I were processed for univariate statistical testing using Student's *t*-test in the Metaboanalyst 5.0 package to compare the relative concentrations of metabolites between cohorts. Data in Figures 2A–C, 3A–C, 4B were presented as box plot showing the minimum, 25th percentile, median, 75th percentile, and maximum. Statistics were analyzed using Graphpad Prism 8.4.3. Differences between these groups were evaluated by one-way ANOVA with Tukey's Honestly Significant Difference Test. *P* < 0.05 was considered statistically significant. Data in Figure 5C were presented as Mean ± SEM. Statistics were analyzed using Graphpad Prism 8.4.3. Differences between these groups were evaluated by one-way ANOVA with Tukey's Honestly Significant Difference Test. *P* < 0.05 was considered statistically significant.

## Results

Heat map of abundance of metabolites from P1, P3, P7, and P28 samples showed the differently regulated metabolites in these samples (Figure 1A, Table 1). Principal component analysis (PCA) revealed that metabolomic profile of P28 animals was clearly separated from the P1, P3 and P7 hearts (Figure 1B). A total of 79 metabolic features changed significantly (*p* < 0.05) in at least one of the group comparisons (P1 vs. P3, P1 vs. P7, and P1 vs. P28) for analyses with LC-MS/MS (Figure 1C). Individual comparison between P1 and P3 hearts didn't reveal a clear separation of metabolomic profiles (Figure 1D), and 14 metabolites were identified with differential expression (Figure 1E). A comparison between P1 and P7 hearts revealed a clear separation of metabolomic profiles (Figure 1F), and 25 metabolites were identified with differential expression (Figure 1G). Finally, a comparison between P1 and P28 hearts revealed a clear separation of metabolomic profiles (Figure 1H), a total of 135 metabolites were detected and 74 metabolites were identified with differential expression. Of the 74 differentially expressed metabolites, abundance of 40 metabolites were increased, and the abundance of 34 metabolites was decreased (Figure 1I).

**Table 1:** List of metabolic features changed in the group comparisons.

	***F*** **value**	***P*** **value**	**−Log10(p)**	**FDR**	**Fisher's LSD**
Proline	254.4	4.31E-16	15.37	5.81E-14	P01–P07; P01–P28; P03–P07; P03–P28; P07–P28
Tyrosine	92.77	6.58E-12	11.18	4.44E-10	P01–P03; P01–P07; P01–P28; P03–P28; P07–P28
Dulcitol	68.41	1.08E-10	9.966	4.16E-09	P01–P28; P03–P28; P07–P28
Sorbitol	66.45	1.41E-10	9.852	4.16E-09	P01–P28; P03–P28; P07–P28
D-Mannitol	65.79	1.54E-10	9.813	4.16E-09	P01–P28; P03–P28; P07–P28
Glycylproline	56.8	5.76E-10	9.24	1.30E-08	P03–P01; P01–P28; P03–P07; P03–P28; P07–P28
Glycocyamine	38.57	1.66E-08	7.779	2.94E-07	P01–P03; P01–P07; P01–P28; P03–P28; P07–P28
Picolinic acid	38.36	1.74E-08	7.758	2.94E-07	P01–P03; P01–P07; P01–P28; P03–P28; P07–P28
Inosine	32.92	6.25E-08	7.204	8.69E-07	P28–P01; P28–P03; P28–P07
Asparagine	32.8	6.44E-08	7.191	8.69E-07	P01–P28; P03–P28; P07–P28
5-Aminolevulinic acid	31.03	1.01E-07	6.994	1.18E-06	P03–P01; P07–P01; P01–P28; P03–P28; P07–P28
Valine	30.91	1.05E-07	6.98	1.18E-06	P01–P07; P01–P28; P03–P28; P07–P28
Hydroxyproline	29.8	1.41E-07	6.852	1.46E-06	P03–P01; P07–P01; P01–P28; P03–P28; P07–P28
Agmatine	28.43	2.06E-07	6.687	1.98E-06	P01–P07; P01–P28; P03–P07; P03–P28; P07–P28
2-Deoxyadenosine	28.04	2.30E-07	6.639	2.07E-06	P28–P01; P28–P03; P28–P07
Cytidine	26.3	3.82E-07	6.418	3.22E-06	P03–P01; P07–P01; P28–P01; P07–P03; P28–P03
Cytosine	25.4	5.03E-07	6.299	3.87E-06	P03–P01; P07–P01; P28–P01; P07–P03; P28–P03; P28–P07
D-Ribose 5-phosphate	25.32	5.16E-07	6.288	3.87E-06	P07–P01; P28–P01; P28–P03; P28–P07
Dimethylglycine	24.24	7.25E-07	6.14	5.15E-06	P01–P28; P03–P28; P07–P28
2-Aminobutyric acid	23.97	7.88E-07	6.104	5.32E-06	P01–P28; P03–P28; P07–P28
Pipecolinic acid	23.51	9.16E-07	6.038	5.89E-06	P03–P01; P03–P07; P03–P28
Oxaloacetic acid	23.32	9.76E-07	6.011	5.99E-06	P01–P28; P03–P28; P07–P28
N-Acetylornithine	22.89	1.13E-06	5.949	6.61E-06	P03–P01; P07–P01; P28–P01; P28–P03; P28–P07
4-Aminobutyric acid	22.62	1.23E-06	5.91	6.92E-06	P01–P07; P01–P28; P03–P07; P03–P28; P07–P28
Norvaline	21.03	2.13E-06	5.671	1.15E-05	P03–P01; P01–P07; P28–P01; P03–P07; P28–P07
Sarcosine	16.38	1.30E-05	4.885	6.76E-05	P03–P01; P01–P28; P03–P07; P03–P28; P07–P28
N-Acetyl-D-galactosamine	15.73	1.73E-05	4.763	8.63E-05	P01–P07; P01–P28; P03–P07; P03–P28; P07–P28
Deoxycytidine	15.41	1.99E-05	4.702	9.12E-05	P07–P01; P07–P03; P28–P03; P07–P28
Hypoxanthine	15.41	1.99E-05	4.701	9.12E-05	P28–P01; P28–P03; P28–P07
Serine	15.37	2.03E-05	4.693	9.12E-05	P01–P28; P03–P28; P07–P28
Dopamine	14.92	2.48E-05	4.605	0.000108	P28–P01; P28–P03; P28–P07
Glucose 6-phosphate	14.81	2.61E-05	4.584	0.00011	P01–P03; P01–P07; P01–P28; P03–P28; P07–P28
Glutamine	14.31	3.28E-05	4.484	0.000134	P28–P01; P28–P03; P28–P07
Quinolinic acid	14.2	3.46E-05	4.461	0.000137	P03–P01; P07–P01; P28–P01; P03–P07; P03–P28
Tryptophan	13.78	4.21E-05	4.376	0.000162	P01–P28; P07–P03; P03–P28; P07–P28
Glucosamine 6-phosphate	13.65	4.49E-05	4.348	0.000168	P01–P28; P03–P28; P07–P28
Alanine	12.55	7.72E-05	4.113	0.000282	P03–P01; P01–P28; P03–P07; P03–P28
Guanosine	12.06	9.95E-05	4.002	0.000353	P07–P01; P28–P01; P28–P03; P28–P07
Betaine	11.81	0.000114	3.945	0.000393	P01–P03; P01–P07; P01–P28
Xanthosine	11.32	0.000147	3.832	0.000497	P07–P01; P28–P01; P28–P03; P28–P07
Uridine	11.03	0.000173	3.763	0.000568	P07–P01; P28–P01; P07–P03; P28–P03
Phenylalanine	9.948	0.000319	3.496	0.001021	P01–P07; P01–P28; P03–P28; P07–P28
9-Octadecynoic acid	9.915	0.000325	3.488	0.001021	P01–P07; P28–P01; P03–P07; P28–P03; P28–P07
5-Hydroxyindoleacetic acid	9.867	0.000334	3.476	0.001026	P01–P28; P03–P28; P07–P28
Asymmetric dimethylarginine	9.779	0.000352	3.453	0.001057	P01–P03; P01–P07; P28–P03; P28–P07
Leucine	9.707	0.000368	3.435	0.001068	P03–P01; P07–P01; P03–P07; P03–P28
Isoleucine	9.688	0.000372	3.43	0.001068	P03–P01; P07–P01; P03–P07; P03–P28
Pyroglutamic acid	9.597	0.000392	3.406	0.001086	P28–P01; P28–P03; P28–P07
Amiloride	9.589	0.000394	3.404	0.001086	P28–P01; P28–P03; P28–P07
Nicotinamide	9.361	0.000452	3.345	0.001198	P28–P01; P28–P03; P28–P07
Suberic acid	9.36	0.000452	3.345	0.001198	P28–P01; P28–P03; P28–P07
Decanoylcarnitine	8.03	0.001046	2.981	0.002715	P28–P01; P28–P03; P28–P07
Adenine	7.858	0.001171	2.931	0.002983	P28–P01; P28–P03; P28–P07
Indole-3-lactic acid	7.697	0.001304	2.885	0.00326	P01–P28; P03–P28; P07–P28
Citrulline	7.227	0.001797	2.746	0.004351	P07–P01; P07–P03; P28–P03; P07–P28
Aspartate	7.22	0.001805	2.744	0.004351	P01–P28; P03–P28; P07–P28
Valeric acid	7.094	0.00197	2.706	0.004666	P01–P28; P03–P28; P07–P28
Methylguanidine	7.054	0.002026	2.693	0.004716	P28–P01; P28–P03; P28–P07
Amino valerate	6.827	0.002379	2.624	0.005443	P01–P03; P01–P07; P01–P28
Adenosyl-L-homocysteine	5.635	0.005752	2.24	0.012942	P07–P01; P07–P03; P28–P03
Taurine	5.285	0.007569	2.121	0.016751	P01–P03; P28–P03
Adenosine	5.119	0.008637	2.064	0.018289	P28–P01; P28–P07
Cellobiose	5.114	0.008676	2.062	0.018289	P01–P28; P03–P28; P07–P28
Lactose	5.113	0.008683	2.061	0.018289	P01–P28; P03–P28; P07–P28
Acetylcholine	5.095	0.008806	2.055	0.018289	P03–P01; P28–P01; P03–P07; P28–P07
Acetylcarnitine	4.993	0.009565	2.019	0.019564	P03–P01; P07–P01
Ribose	4.681	0.012356	1.908	0.024896	P28–P01; P28–P03; P28–P07
2-Hydroxyphenylacetic acid	4.614	0.013069	1.884	0.025945	P01–P28; P03–P07; P03–P28
Xylose	4.525	0.014069	1.852	0.027527	P28–P01; P28–P03; P28–P07
3-hydroxybutyric acid	4.461	0.014851	1.828	0.028641	P01–P03; P01–P07; P28–P03; P28–P07
PEP	4.4	0.015639	1.806	0.029737	P01–P28; P07–P03; P07–P28
Trehalose	4.296	0.01709	1.767	0.03172	P01–P28; P03–P28
Lactate	4.292	0.017152	1.766	0.03172	P28–P01; P28–P03; P28–P07
NADPH	4.265	0.017552	1.756	0.03202	P28–P01; P07–P03; P28–P03
2-Deoxyuridine	4.167	0.019096	1.719	0.034373	P03–P01; P07–P01; P28–P01
Acetylglucosamine	3.881	0.024523	1.61	0.043006	P01–P28; P03–P28
Homoserine	3.866	0.024848	1.605	0.043006	P07–P01; P07–P03; P07–P28
Threonine	3.866	0.024848	1.605	0.043006	P07–P01; P07–P03; P07–P28
ADP	3.725	0.028165	1.55	0.048129	P01–P03

Nucleotide biosynthesis requires ribose, glycine, aspartate, and glutamine (7). Our data indicated a decreased abundance of glycine and aspartate (Figure 2A) and an increased abundance of several intermediate metabolites (guanosine, adenosine, inosine, hypoxanthine, and ADP) in purine degradation pathways (Figure 2B) in P28 comparing to P1 hearts. This data is consistent with our previous observation that cardiomyocyte cell cycle is significantly reduced in P28 comparing to P1 hearts. Interestingly, abundance of ribose and ribose-5-phosphate was significantly increased in P28 comparing to P1 hearts (Figure 2A). Ribose as its 5-phosphate ester (ribose-5-phosphate) is typically produced from glucose by pentose phosphate pathway. Our data from the pathway analysis of glucose metabolism suggested that, while the intracellular glucose level remained unchanged among P1, P3, P7, and P28 hearts, abundance of the intermediate metabolites in the pathways of glycolysis [glucose-6-phosphate, Phosphoenolpyruvate (PEP), and 3-phosphoglyceric acid] were decreased in P28 comparing to P1 hearts (Figure 2C), which implicates an overall reduced level of glycolysis. The decreased abundance of oxaloacetic acid and increased abundance of lactic acid in P28 hearts suggested that glycolysis rather than glucose oxidation is the main form of glucose metabolism in mature pig hearts (Figure 2C). Reduced level of the metabolism of lactose, sorbitol, and dulcitol (galactitol) was also observed, which implicates a reduced availability of the storage form of glucose when pig heart matures. Abundance of fructose remained stable during the postnatal heart development, and abundance of raffinose showed a tendency of reduction though it didn't reach the significant level (Figure 2C).

The abundance of glucogenic amino acids (alanine, asparagine, valine, and serine) significantly reduced in P28 comparing to P1 hearts, while no change was found for abundance of glutamic acids and histidine (Figure 3A). The biological implication of these changes to heart development is not clear since gluconeogenesis occurs mainly in liver and rarely in heart muscles. Abundance of leucine, a ketogenic amino acid, increased in P3 hearts followed by a tendency of reduction in P7 and P28 hearts (Figure 3B), implicating increased keto metabolism in early postnatal pig hearts. Abundance of ketogenic and glucogenic amino acids (phenylalanine, tyrosine, and tryptophan) were constantly reduced from P1 to P28 hearts. Abundance of isoleucine was increased in P3 hearts but gradually decreased in P7 and P28 hearts. In addition, abundance of threonine was increased in P7 followed by a reduction in P28 hearts (Figure 3C).

The abundance of glucosamine 6-phosphate (GLCN6P) was reduced in P28 hearts comparing to P1 hearts (Figure 3D). Fructose 6-phosphate and glutamine are catalyzed by glutamine-fructose 6-phosphate amidotransferase to form GLCN6P, which is then converted to UDP-N-acetylglucosamine (UDP-GlcNAC) through a series of enzyme-driven reactions (8). UDP-GlcNAc is an important posttranslational protein modification on Ser/Thr sites driven by O-GlcNAc transferase (OGT) and this modification has been shown to contribute to cardiomyocyte cell cycle regulation in mouse hearts (9). However, the role of this posttranslational modification (O-glycosylation) in pig hearts has not been reported.

Collagen triple helix is one of the few proteins that contain the amino acid hydroxyproline, therefore, hydroxyproline has been used to estimate total collagen content and concentration in mouse and human tissues (10, 11). It was reported that collagen content increases constantly in developing pig hearts during the first 8 weeks (12). In human hearts, the total amount of collagen increases with age (10). However, the ratio of total collagen to total protein is high after birth and it gradually declines during development, with the total collagen/total protein ratio reaching normal levels around 5 months after birth (10). In the current study, abundance of all metabolites was normalized by total protein content. The abundance of hydroxyproline was high in P1, P3, and P7 hearts comparing to the P28 heart (Figure 3E). This data suggests a high ratio of collagen to total protein in the young pig hearts which is consistent with the observations from human hearts.

Functional batch annotation and ontology enrichment analysis identified important representative metabolite ontology categories based on KEGG pathway. The highly enriched metabolites were found in the amino acid and protein metabolism pathways (Figure 4A). Along with the increased body weight (Figure 4B), the average heart weight of the neonatal pigs was 17.82 ± 1.94 (for P1), 19.63 ± 4.41 (for P3), 20.61 ± 2.44 (for P7), and 48.39 ± 5.32 (for P28) grams, indicating an rapidly increased mass between postnatal day 7 and 28 (Figure 4B). We have previously reported that cardiomyocyte cell cycle activity in neonatal pig hearts declines abruptly (i.e., by ~70%) in the first 7 days after birth. Taken together, these data implicate a massive hypertrophic growth between P7 and P28 when cardiomyocytes withdrawal from cell cycle. The proliferating cells typically require massive anabolic program to generate biological macromolecules such as proteins and nucleotides. Therefore, the overall reduced abundance of different amino acids in P28 comparing to P1 hearts (Figures 3A–E) likely reflects an increased biosynthesis of proteins to accommodate the needs for cell proliferation in P1 hearts, while protein synthesis declines in P28 when proliferation stops.

## Discussion

Our data suggested an active anabolism of nucleotide and proteins in the neonatal pig hearts when cardiomyocytes retain some cell cycle activities. Reduced ratio of total collagen to total protein, active posttranslational protein modification, and a metabolic switch from glucose to fatty acids were also observed when cardiomyocytes become mature. To our knowledge, this is the first study to determine metabolic profile in developing pig hearts.

An important finding of current study is the discovery of the transition from carbohydrate to fatty acid metabolism during postnatal heart development (Figure 2C). The metabolic switch from glycolysis to phosphorylated oxidation of fatty acids in the postanal hearts has been reported in other mammals (13, 14). The gradually increased abundance of acetylcarnitine in P3 and P7 hearts comparing to P1 hearts likely reflects an increased level of fatty acid β-oxidation when cardiomyocytes become mature. Another interesting finding is that the abundance of several metabolites in extracellular matrix (ECM) remodeling was changed from P1 to P28. For example, a reduced abundance of GLCN6P was observed in P28 hearts compared to P1 hearts (Figure 3D). GLCN6P is converted to UDP-GlcNAC through a series of enzyme-driven reactions (8). UDP-GlcNAc is an important substrate for the synthesis of proteoglycans, hyaluronan, glycolipid, et al. (15). We also observed an increased abundance of hydroxyproline in P1, P3, and P7 hearts compared to the P28 heart (Figure 3E). Hydroxyproline has been used as a marker for estimating total collagen content and concentration in mouse and human tissues (10, 11). Taken together, our data suggest an active ECM remodeling in the postnatal pig heart.

Despite the reduced abundance of intermediates in glycolysis pathway, the abundance of lactic acid was increased in P28 pig hearts compared to P1 pig hearts (Figure 2C). This data are consistent with the observation that lactic acid is gradually increased in postnatal mouse hearts (4). Intracellular lactic acid can be produced from pyruvate during the last step of glycolysis which is a reversible conversion catalyzed by lactate dehydrogenases (LDHs) (16), or be obtained from peripheral circulation by monocarboxylic acid cotransporters (MCTs) (17, 18). LDHs are homo- or hetero-tetramers assembled from two different subunits (M and H), which are encoded by two separate genes, LDHA (M) and LDHB (H), respectively (19). LDH1 is composed of four LDHB subunits and favors the conversion from lactate to pyruvate, and LDH5 is composed of four LDHA subunits and favors the conversion from pyruvate to lactate (20). Expression of LDHA is induced during physiological (e.g., running and swimming) and pathological (e.g., pressure overload induced by thoracic aortic constriction) cardiac hypertrophy (21, 22). We performed western blot experiments to check the expression of LDHs in the postnatal pig hearts. Our data showed that expression of LDHA was decreased and LDHB was increased in P28 hearts comparing to P1, P3, and P7 hearts (Figures 5A–C). During cardiac maturation, lactic acid oxidation and glycolysis were reduced, and fatty acid oxidation became the main energy supply (14). However, the ratio of LDH to LDHA continued to rise during the first month after birth in guinea pigs (23), which is consistent with our observations in postnatal pig hearts. Detailed mechanisms underlying the changes of LDHA and LDHB in postnatal hearts were not clear. Considering that glycolysis is reduced in P28 hearts in the current study, we speculate that the increased lactic acid is likely due to increased uptake from peripheral blood.

It is worthy to note that time window for these metabolic switch matches the window that neonatal pig hearts lose their regeneration capacity post injury (2). Recent data showed that simulating the postnatal switch in metabolic substrates from carbohydrates to fatty acids promotes cell cycle arrest in human cardiomyocytes (24). However, it was also reported that stimulation of glycolysis promotes cardiomyocyte proliferation in zebrafish (25). Thus, far, it is not clear if the metabolic switches we observed in postnatal pig hearts contribute to cardiomyocyte cell cycle arrest. Future studies are warranted to explore whether manipulating metabolic pathways enhances myocardial regeneration and repair capacities in neonatal and adult pigs.

The current study has several limitations. First, the whole heart samples were used for this study. Although the mass or volume of cardiomyocytes dominates in the heart, non-myocytes are more abundant than cardiomyocytes. The challenges of cultivating the freshly isolated pig cardiomyocytes precludes the uses of highly purified cardiomyocytes for LC-MS/MS analysis. Second, this is a descriptive study investigating the abundances of different metabolites at different stages of postnatal heart development (P1, P3, P7, and P28) in pigs. The evaluation of activity of certain metabolic pathways was based on the abundances of different metabolites in these pathways. Further studies, e.g., metabolic flux measurements, enzyme activity assays, and uptake of metabolites, are warranted to confirm the data from the metabolomic analysis.

## Data Availability Statement

The datasets presented in this study can be found in online repositories (https://data.mendeley.com; DOI: 10.17632).

## Ethics Statement

The animal study was reviewed and approved by Institutional Animal Care and Use Committee of the Mayo Clinic.

## Author Contributions

PL, FL, LT, WZha, and YJ collected data and performed data analysis. HG and WZhu designed the project and wrote the manuscript. PL and FL revised the manuscript. All authors contributed to the article and approved the submitted version.

## Funding

This work was supported by NIH grant (R01HL142627) and AHA TPA Award (20TPA35490001) to WZhu.

## Conflict of Interest

The authors declare that the research was conducted in the absence of any commercial or financial relationships that could be construed as a potential conflict of interest.

## Publisher's Note

All claims expressed in this article are solely those of the authors and do not necessarily represent those of their affiliated organizations, or those of the publisher, the editors and the reviewers. Any product that may be evaluated in this article, or claim that may be made by its manufacturer, is not guaranteed or endorsed by the publisher.
